# Supplementary material for: Repurposing host-guest chemistry to sequester virulence and eradicate biofilms in multidrug resistant Pseudomonas aeruginosa and Acinetobacter baumannii
Source: Nat Commun. 2023 Apr 14;14:2141. doi: 10.1038/s41467-023-37749-6 (PMC10104825; doi:10.1038/s41467-023-37749-6)
Supplement: Supplementary file 3 — Description of Additional Supplementary Files [file 41467_2023_37749_MOESM3_ESM.pdf]

Description of Additional Supplementary Files

Supplementary Data 1: Clinical isolates used in this study.
